# The Fish Pathogen “*Candidatus* Clavichlamydia salmonicola”—A Missing Link in the Evolution of Chlamydial Pathogens of Humans

**DOI:** 10.1093/gbe/evad147

**Published:** 2023-08-24

**Authors:** Astrid Collingro, Stephan Köstlbacher, Alexander Siegl, Elena R Toenshoff, Frederik Schulz, Susan O Mitchell, Thomas Weinmaier, Thomas Rattei, Duncan J Colquhoun, Matthias Horn

**Affiliations:** Centre for Microbiology and Environmental Systems Science, University of Vienna, Vienna, Austria; Centre for Microbiology and Environmental Systems Science, University of Vienna, Vienna, Austria; Doctoral School in Microbiology and Environmental Science, University of Vienna, Vienna, Austria; Laboratory of Microbiology, Wageningen University and Research, Wageningen, The Netherlands; Centre for Microbiology and Environmental Systems Science, University of Vienna, Vienna, Austria; Centre for Microbiology and Environmental Systems Science, University of Vienna, Vienna, Austria; Institute of Molecular Biology and Biophysics, Eidgenössische Technische Hochschule Zürich (ETH), Zürich, Switzerland; Centre for Microbiology and Environmental Systems Science, University of Vienna, Vienna, Austria; DOE Joint Genome Institute, Berkeley, California, USA; FishVet Group Ireland, Oranmore, Co. Galway, Ireland; Centre for Microbiology and Environmental Systems Science, University of Vienna, Vienna, Austria; Centre for Microbiology and Environmental Systems Science, University of Vienna, Vienna, Austria; Department of Biological Sciences, University of Bergen, Bergen, Norway; Centre for Microbiology and Environmental Systems Science, University of Vienna, Vienna, Austria

**Keywords:** Clavichlamydia, chlamydiae, fish pathogen, *Chlamydia trachomatis*, gene families

## Abstract

Chlamydiae like *Chlamydia trachomatis* and *Chlamydia psittaci* are well-known human and animal pathogens. Yet, the chlamydiae are a much larger group of evolutionary ancient obligate intracellular bacteria that includes predominantly symbionts of protists and diverse animals. This makes them ideal model organisms to study evolutionary transitions from symbionts in microbial eukaryotes to pathogens of humans. To this end, comparative genome analysis has served as an important tool. Genome sequence data for many chlamydial lineages are, however, still lacking, hampering our understanding of their evolutionary history. Here, we determined the first high-quality draft genome sequence of the fish pathogen “*Candidatus* Clavichlamydia salmonicola”, representing a separate genus within the human and animal pathogenic Chlamydiaceae. The “*Ca.* Clavichlamydia salmonicola” genome harbors genes that so far have been exclusively found in *Chlamydia* species suggesting that basic mechanisms important for the interaction with chordate hosts have evolved stepwise in the history of chlamydiae. Thus, the genome sequence of “*Ca.* Clavichlamydia salmonicola” allows to constrain candidate genes to further understand the evolution of chlamydial virulence mechanisms required to infect mammals.

**Significance Statement**

Chlamydiae is a diverse group of intracellular bacteria, but apart from the well-known human and animal pathogens, and protist symbionts, knowledge about most chlamydial lineages is scarce. We analyzed the first genome sequence of a fish-pathogenic member of the Chlamydiaceae, representing the genus Clavichlamydia, and we show that important chlamydial virulence genes including protein domains for interaction with host proteins have emerged in a stepwise manner during the evolution of Chlamydiaceae. These genes might have contributed to the transition of chlamydiae to successfully infect higher animals in the past. Thus, the genome sequence of “*Candidatus* Clavichlamydia salmonicola” provides an important piece in the puzzle towards understanding the evolution of chlamydiae from bacterial symbionts of protists to major human pathogens.

## Introduction

Chlamydiae are a large phylum of obligate intracellular bacteria infecting a broad spectrum of eukaryotic hosts ranging from protists to invertebrates and mammals (Taylor-Brown et al. 2015; Collingro et al. 2020). Members of six chlamydial families, namely the Piscichlamydiaceae, Parilichlamydiaceae, Clavichlamydiaceae/Chlamydiaceae, Parachlamydiaceae, Simkaniaceae, and Rhabdochlamydiaceae are able to infect a variety of fish, where they are—together with a few other bacteria—associated with the poorly understood gill and skin disease epitheliocystis (Toenshoff et al. 2012; Stride et al. 2014; Katharios et al. 2015; Pawlikowska-Warych and Deptuła 2016; Seth-Smith et al. 2016; Blandford et al. 2018). Members of the Piscichlamydiaceae, Parilichlamydiaceae, and *Clavichlamydia* are particularly prevalent. They seem to be restricted to fish as they have not been detected in other hosts so far. Any attempts to culture these bacteria in surrogate host systems in the lab have failed so far (Blandford et al. 2018). “*Candidatus* Clavichlamydia salmonicola” (hereafter referred to as *Cl. salmonicola*), infects farmed and wild salmonids in freshwater and often co-occurs with *Piscichlamydia* species (Rourke et al. 1984; Bradley et al. 1988; Karlsen et al. 2008; Mitchell et al. 2010; Schmidt-Posthaus et al. 2012; Guevara Soto et al. 2016a, 2016b, 2017).

*Candidatus Cl. salmonicola* represents an early branching genus within the Chlamydiaceae, which includes important human and animal pathogens like *Chlamydia trachomatis, Chlamydia pneumoniae,* and *Chlamydia psittaci* (Elwell et al. 2016). Since attempts to cultivate *Cl. salmonicola* in fish and insect cell lines as well as amebae were not successful, and as publicly available metagenomic studies have not captured clavichlamydiae so far (Köstlbacher et al. 2021b), we here applied a cultivation-independent, targeted metagenomic approach to obtain insights into the genetic repertoire of this chlamydial species. In order to study common chlamydial virulence mechanisms in vertebrate hosts, that is to identify genes involved in host adaptation, host-specificity, and virulence, we then compared the *Cl. salmonicola* genome with other chlamydial genomes with a focus on the closely related members of the Chlamydiaceae and the recently proposed family Sororchlamydiaceae (Dharamshi et al. 2020, 2022) as well as the only other fish pathogens with available genome sequences, members of the Parilichlamydiaceae (Taylor-Brown et al. 2017, 2018).

## Results and Discussion

### General Genome Features

Total genomic DNA was extracted from infected gill tissue of Atlantic salmon (*Salmo salar*) from a commercial fish farm in the northwest of Ireland in February 2009 (Mitchell et al. 2010) and sequenced using a combination of pyrosequencing and Illumina technologies.

The assembly of the *Cl. salmonicola* genome consists of 28 scaffolds with a total length of 1,392,990 bp (25 × coverage) and a G+C content of 32.48% (supplementary table S1, Supplementary Material online). According to analysis with CheckM2 v0.1.3 (Chklovski et al. 2022), the genome assembly has a completeness of 94.68% with 0.27% contamination, suggesting a theoretical genome size for *Cl. salmonicola* of roughly 1.47 Mbp. The assembly includes a complete rRNA gene operon on the largest 470,378 nt scaffold, as well as 37 tRNA genes, and 1,105 protein-coding sequences (CDS). One of the scaffolds (contig 16) represents a plasmid of 8,040 bp (9 CDS, G+C content 28%), which is highly similar to the small plasmids of other Chlamydiaceae species (supplementary table S1, Supplementary Material online, supplementary fig. S1, Supplementary Material online) (Köstlbacher et al. 2021a).

### Clavichlamydia Salmonicola Represents a Novel Genus Within the Chlamydiaceae

Previous 16S rRNA gene-based phylogenies consistently suggested *Cl. salmonicola* as representative of the family-level lineage Clavichlamydiaceae and being the closest relative of the human and animal pathogenic Chlamydiaceae (Karlsen et al. 2008; Stride et al. 2014). The 16S rRNA gene sequence present in the *Cl. salmonicola* genome is almost identical (99.68%) to other publicly available clavichlamydial 16S rRNA gene sequences (supplementary fig. S2, Supplementary Material online), but the nucleotide identity of 90.14–91.86% to *Chlamydia* species and 91.91–92.1% to members of the recently described genus *Chlamydiifrater* (Vorimore et al. 2021) suggests *Clavichlamydia* to represent a genus within the Chlamydiaceae family instead. This is further corroborated by the analysis of average nucleotide identities (ANI) and average amino acid identities (AAI) between the genomes of *Cl. salmonicola* and other chlamydiae (supplementary table S2, Supplementary Material online). ANI values of the *Clavichlamydia* genome to those of the genera *Chlamydiifrater* and *Chlamydia* (65.67–65.82% and 65.39–66.37%, respectively) are in a similar range as between *Chlamydiifrater* and *Chlamydia* genomes (65.7–66.2%) (Vorimore et al. 2021). While ANI serve well for species delineation, AAI are better suited to resolve genus- and family-level clades (Konstantinidis et al. 2017). The AAI between *Cl. salmonicola* and other Chlamydiaceae genomes are with 47.1–48.2% (alignment fraction >60%) within the family-level boundary of 45–65% (Konstantinidis et al. 2017), whereas they are lower to all other chlamydial genomes analyzed (supplementary table S2, Supplementary Material online). Thus, we propose to reclassify *Cl. salmonicola* as the single representative of the genus *Clavichlamydia* within the Chlamydiaceae rather than representing a family-level lineage.

**Fig. 1:**
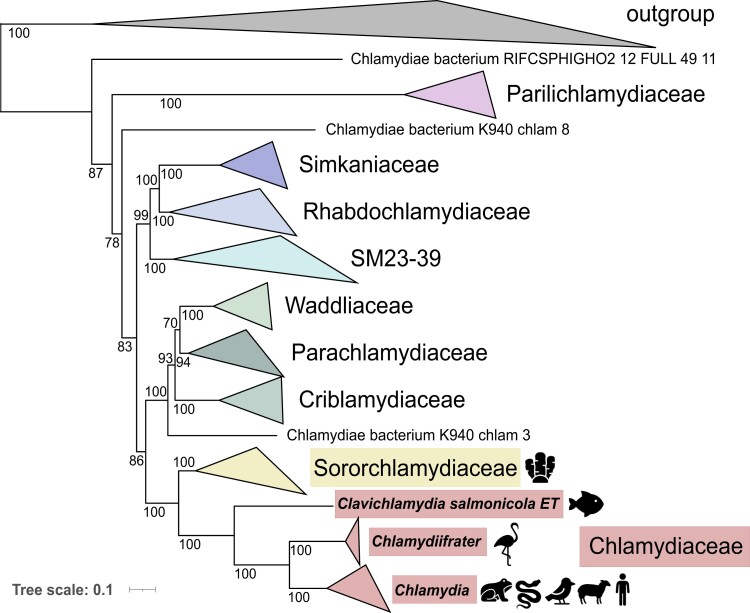
*Clavichlamydia salmonicola* is most closely related to other genera in the Chlamydiaceae and the Sororchlamydiaceae. Genome phylogeny using publicly available chlamydial genome sequences as well as Planctomycetes and Verrucomicrobia sequences as an outgroup (supplementary table S2, Supplementary Material online). Maximum likelihood tree inference was performed with 43 concatenated CheckM single copy marker protein sequences using IQ-TREE (-TESTNEW -PMSF -b 100). Nonparametric bootstrap values are indicated in the tree. Family-level groups were collapsed. A complete phylogenetic tree including the outgroup is available as supplementary figure S3, Supplementary Material online. The initial tree serving as guide tree for the PMSF modeled tree including ultrafast bootstrap values and SH-like approximate likelihood ratio test values (IQ-TREE -TESTNEW -bnni 1000 -alrt 1000) is available as supplementary figure S4, Supplementary Material online. For Sororchlamydiaceae and Chlamydiaceae, representative host organisms from the respective phylum or vertebrate class are depicted (illustrations have been downloaded from Flaticon.com).

To further confirm the phylogenetic relationship of *Cl. salmonicola* with Chlamydiaceae and other chlamydial lineages, especially those that are mainly represented by metagenome-assembled genomes (MAGs) and often lack a 16S rRNA gene, we inferred a maximum likelihood species tree based on the concatenated alignment of 43 marker genes (supplementary table S3, Supplementary Material online) (Parks et al. 2015). Our analysis shows that *Clavichlamydia* is a sister lineage to *Chlamydiifrater* and *Chlamydia*, and Chlamydiaceae are monophyletic with a clade represented until very recently only by MAGs derived from deep-sea sediments and an ant-fungus garden (fig. 1, Supplementary figs. 3 and 4, Supplementary Material online) (Dharamshi et al. 2020; Köstlbacher et al. 2021b). In a new study, four additional sponge-associated MAGs were published, and the name Sororchlamydiaceae was proposed for this clade (Dharamshi et al. 2022). Phylogenetic analyses in our study and comprehensive phylogenetic analyses of the chlamydial phylum in a recently published study further corroborate the phylogenetic placement of *Clavichlamydia* close to *Chlamydia* (Dharamshi et al. 2022, 2023). Phylogenies inferred for the 16S rRNA gene as well as the single copy marker genes show that sponge-associated chlamydiae are basal to fish (*Clavichlamydia*), amphibian (*Amphibiichlamydia*, only represented by 16S rRNA gene data so far), bird (*Chlamydiifrater*), and amphibia/bird/reptile/mammal (*Chlamydia*) infecting chlamydial genera (fig. 1, Supplementary figs. 2–4, Supplementary Material online) (Borel et al. 2018).

### Virulence-Associated Genes Shared Amongst Chlamydial Lineages

The obligate intracellular chlamydial lifestyle is ancient and shared by all known chlamydiae (Collingro et al. 2020). Their biphasic developmental cycle alternates between an extracellular infectious stage, the elementary body, and an intracellular replicative form, the reticulate body (Elwell et al. 2016). Chlamydiae employ a type III secretion (T3S) system, which has already been present in the last common chlamydial ancestor, and effector proteins as the main toolbox to infect and interact with their eukaryotic host cells (Peters et al. 2007; Mueller et al. 2014; Dharamshi et al. 2023). After host cell entry, chlamydiae usually reside within a host-derived vacuole called inclusion (Elwell et al. 2016). Various chlamydial proteins are present in the inclusion lumen or inserted into its membrane (Bugalhão and Mota 2019; Gitsels et al. 2019).

**Fig. 2:**
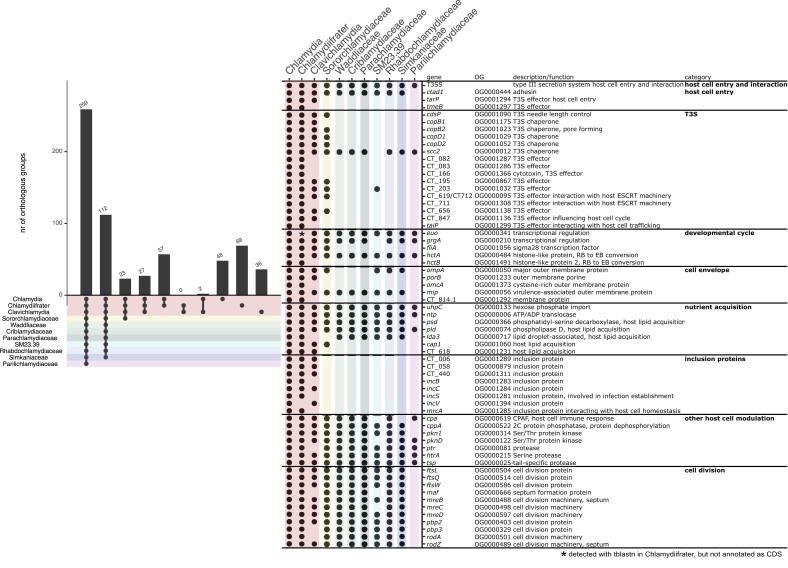
Genes shared between Chlamydiaceae and other chlamydiae. Presence and absence of gene families in genomes of members of known chlamydial families. Chlamydial family-level lineages are color-coded as in figure 1. In the left panel, the presence of orthologous gene families is indicated by black circles. The right panel shows presence and absence of selected genes in genomes of members of chlamydial families. Gene names, annotations, and the broader functional category are indicated.

Our analysis of orthologous groups (OGs) of proteins in 49 chlamydial genomes confirmed the presence of well-conserved genes indicative of the basic host-dependent chlamydial lifestyle in *Cl. salmonicola* (fig. 2) (Köstlbacher et al. 2021b). These genetic traits include the T3S system and various effector proteins, genes for host cell adhesion, acquisition of host nutrients, proteases and kinases to modulate the host cell, and major transcriptional regulators of the developmental cycle (fig. 2).

*Chlamydia* species encode a number of additional gene families that so far have not been detected in any other chlamydiae and have been associated with virulence in humans and animals. With the availability of MAGs from Sororchlamydiaceae and genomes of the novel genera *Clavichlamydia* and *Chlamydiifrater* it is now possible to analyze differential presence of these genes within the Chlamydiaceae and their sister family. Interestingly, 23 of these gene families so far exclusively present in Chlamydiaceae are also found in Sororchlamydiaceae genomes (fig. 2). Among them are some genes that have previously been shown in *Chlamydia* species to be important for host cell interaction. This includes the T3S system needle length determinator CdsP (Lorenzini et al. 2010), and the chaperones CopB2, CopD2, and CopD1, which are located at the tip of the T3S system needle and considered to be essential for infection of nonphagocytic host cells (Matteï et al. 2011; Bulir et al. 2014). Some other T3S effectors (CT_195 and CT_656) some of which interact with the host cell's endosomal sorting complexes required for transport (ESCRT) machinery (CT_619, CT_712; Vromman et al. 2016) as well as inclusion proteins involved in the acquisition of host cell lipids (Cap1) are shared too (fig. 2) (Bugalhão and Mota 2019). The transcription of many genes expressed late during the developmental cycle is regulated by σ^28^ in *Chlamydia* species (Yu et al. 2006). This transcription factor is also encoded in sponge-associated Sororchlamydiaceae and all Chlamydiaceae genomes, whereas it is absent in other chlamydiae (fig. 2) (Domman and Horn 2015). A recent study modeling ancestral chlamydial genomes and their evolution showed that the above-mentioned genes with the exception of σ^28^ appeared first in the last common ancestor of Sororchlamydiaceae and Chlamydiaceae, but their origin is unknown (Dharamshi et al. 2023) (supplementary table S4, Supplementary Material online). The acquisition of these genes might have primed members of these chlamydial lineages for the infection of metazoan hosts.

Gene families exclusively present in all Chlamydiaceae (*n* = 27) include additional T3S genes like *copB1* and T3S effectors important for the entry of nonphagocytic host cells (*tarP*) (Jewett et al. 2006; Ghosh et al. 2020), potentially modulating the host cell cycle (CT_847; Elwell et al. 2016), host lipid acquisition (CT_618), or inclusion proteins (CT_006, CT_058, CT_440, *incC*; Bugalhão and Mota 2019) (fig. 2). Again, apart from *tarP* (see below), these gene families have first occurred in the ancestor of Chlamydiaceae, and their initial source is unknown as there are no known homologs in current sequence databases (Dharamshi et al. 2023) (supplementary table S4, Supplementary Material online).

Chlamydiaceae also harbor a small plasmid (8–9 kb), which is highly conserved in gene synteny (Supplementary fig. 1, Supplementary Material online). The *Cl. salmonicola* plasmid encodes all eight genes known from *Chlamydia* plasmids (30.1–58.9%, mean 39.9% amino acid identity) and an additional partial DNA helicase. Two of the genes on the chlamydial plasmid (*pgp3, pgp4*) were previously considered to be specific to members of the genus *Chlamydia* but are indeed found on all Chlamydiaceae plasmids. These genes have been implicated in chlamydial niche differentiation towards higher animals, and they have recently been proposed to be involved in the formation of putative outer membrane vesicles delivering proteins into the host cytosol (Köstlbacher et al. 2021a; Lei et al. 2021).

Within Chlamydiaceae, there are 57 orthologous genes exclusively shared by members of the genera *Chlamydiifrater* and *Chlamydia* (fig. 2). This includes the membrane proteins OmcA and CT_814.1, the histone-like protein HctB, and T3S effectors affecting inclusion growth (*taiP*; Hamaoui et al. 2020) and interacting with the host ESCRT machinery (CT_711; Bugalhão and Mota 2019) (fig. 2, supplementary table S4, Supplementary Material online). Again, some inclusion proteins were gained for potential host interaction and modulation including *incB*, *incS*, CT_082 and CT_083, and *mrcA* promoting inclusion extrusion and interfering with host cell homeostasis (Nguyen et al. 2018; Chamberlain et al. 2022). Of note, no gene is shared solely between *Chlamydiifrater* and *Clavichlamydia,* and only two genes are exclusively present in *Chlamydia* and *Clavichlamydia* genomes. One of them representing *incV* important for directing the endoplasmic reticulum towards chlamydial inclusions (Ende et al. 2022). Finally, of the many orthologous genes previously only known to exist in *Chlamydia* species still 48 are specific for members of this genus, potentially reflecting genes necessary for the interaction with bird, reptile, and mammalian cell types or tissues.

Taken together, the in depth-analysis of orthologous genes shared between different chlamydial lineages, especially those shared between and amongst Sororchlamydiaceae and genera in the Chlamydiaceae, revealed major differences in genes involved in host interaction and manipulation. Our findings suggest a sequential acquisition of these genes from *Sorochlamydia*-like ancestors infecting basal animal clades such as sponges to Clavichlamydiae infecting fish; and further from *Chlamydiifrater* infecting birds to *Chlamydia* species infecting amphibians, reptiles, birds, and mammals.

### Evolution of the Chlamydial T3S Effector translocated actin-recruiting effector

**Fig. 3:**
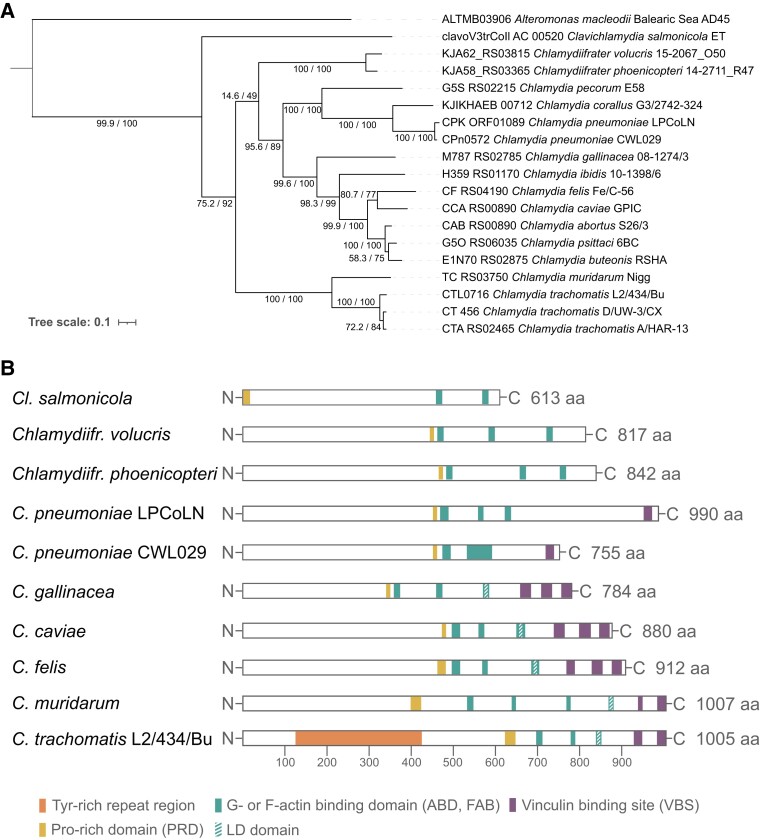
Evolution of TarP in Chlamydiaceae. (*A*) TarP phylogeny including protein sequences from Chlamydiaceae and OMA orthologs (Altenhoff et al. 2021) indicates horizontal acquisition of this gene from Pseudomonadota in the last common Chlamydiaceae ancestor and subsequent maintenance and diversification within the family. Maximum likelihood tree inference using the PMB+F+G4 substitution model (IQ-TREE -TESTNEW -bnni 1000 -alrt 1000). The phylogenetic tree is midpoint rooted. The relevant subtree is shown; the complete version is available as supplementary figure S5, Supplementary Material online. (*B*) Presence of protein domains in chlamydial TarP proteins. Different domains are color-coded and the length of each protein is given in amino acids (aa).

The translocated actin-recruiting effector (TarP) is a well-described key virulence factor necessary for the entry of nonphagocytic host cells (reviewed in Caven and Carabeo 2020), which is specific to members of the Chlamydiaceae (fig. 2). Although TarP has no orthologs in the eggNOG database (Dharamshi et al. 2023), it forms an orthogroup in OMA orthologs (Altenhoff et al. 2021) with 28 distantly related genes from nonchlamydial organisms (supplementary table S4, Supplementary Material online). Phylogenetic analysis of this orthogroup indicates that TarP was acquired by the last common ancestor of the Chlamydiaceae (fig. 3*A*). Within the family, the *Clavichlamydia* TarP represents the deepest branching lineage. The genera *Chlamydiifrater* and *Chlamydia* are not well-separated due to the lack of resolution in this tree, but within both genera, the gene tree is congruent with the species tree, together suggesting maintenance and further diversification of TarP within the Chlamydiaceae. It has been shown previously that TarP phylogeny in *C. trachomatis* correlates with tissue tropism (Lutter et al. 2010).

The recent progress of methods in genetic manipulation of chlamydiae has enabled a better understanding of TarP and its interaction with diverse host factors (Caven and Carabeo 2020). Although the functional domains of TarP have been characterized mainly in *C. trachomatis*, their existence in *C. pneumoniae* has recently also been verified experimentally (Braun et al. 2019). Notably, these domains are absent in the closest related nonchlamydial orthologs of TarP (i.e., in *Alteromonas macleodii*).

A more detailed analysis of the domain architecture of chlamydial TarP revealed an interesting pattern of domain acquisition (fig. 3*B*). All chlamydial TarP proteins have a proline-rich domain (PRD) and at least two G- or F-actin binding domains (ABD or FAB, respectively). PRD is responsible for TarP oligomerization in the host cell, followed by the formation of a G-actin nucleus via the concentrated localization of ABD and subsequent actin polymerization. This eventually results in the remodeling of the host cell cytoskeleton and a phagocytosis-like internalization of chlamydial elementary bodies (Caven and Carabeo 2020). While a single ABD is sufficient for host cell entry (Jewett et al. 2010; Parrett et al. 2016), members of the genus *Chlamydia* have adopted additional ways to recruit host actin with TarP. Their TarP proteins harbor vinculin-binding domains (VBD, *n* = 1–3), which account as a second path to indirectly polymerize F-actin at the chlamydial entry site (Thwaites et al. 2015). A further step in *Chlamydia* adaptation to remodel actin—but missing in *C. pneumoniae—*is via domains rich in leucine and aspartic acid (LD-domains) located in FABs (Thwaites et al. 2014). Finally, TarP of *C. trachomatis* possesses a tyrosine-rich repeat region, which not only plays a role in actin polymerization but also potentially affects the host cell cycle (Shehat et al. 2021). Recently, a postinvasion function of TarP has been discovered, in which LD and VBD domains inhibit cell motility by binding focal adhesion kinase and vinculin, which leads to less cell shedding in epithelial tissue (Pedrosa et al. 2020). This could represent a mechanism of chlamydiae to maintain infections in high-turnover tissues (Pedrosa et al. 2020). Taken together, the acquisition of novel domains in TarP might reflect stepwise adaptations within Chlamydiaceae to refine their potential to manipulate their host cells.

A similar pattern of domain architecture evolution can be observed in the inclusion membrane protein IncV (shared between *Clavichlamydia* and *Chlamydia* species; fig. 2, supplementary table S4, Supplementary Material online). This protein establishes inclusion membrane contact sites (MCS) with the endoplasmic reticulum for the acquisition of host lipids (Ende et al. 2022). While the N-terminal part of the protein is well-conserved in its amino acid sequence and predicted transmembrane helices in *Cl. salmonicola*, the protein is shorter than in *Chlamydia* species and lacks all characterized motifs interacting with mammalian cells to establish the MCS-like phosphorylation sites, FFAT motifs, and VAMP-associated proteins recruitment sites (Ende et al. 2022). Other Chlamydiaceae virulence genes, however, do not differ in motifs or domains and are therefore likely functionally conserved since their origin. CT_712 and CT_619, for instance, T3S effectors interacting with the host ESCRT machinery contain an N-terminal coiled-coil domain for binding host TSG101 and a C-terminal DUF582 domain (Pfam PF04518) in all orthologs (Vromman et al. 2016). In summary, the detailed analysis of domain architecture provides valuable insights into the evolution of chlamydial effector proteins and suggests domain acquisition as a potential mechanism of sequential evolution of host interaction, and eventually host-specificity and tissue tropism.

### The Chlamydial Plasticity Zone

**Fig. 4:**
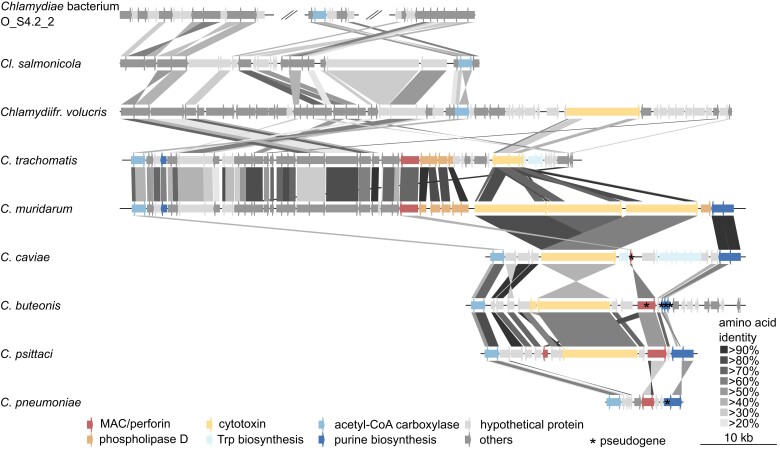
The chlamydial plasticity zone. Genetic organization of syntenic regions in Sororchlamydiaceae and Chlamydiaceae plasticity zones. Several genes of the plasticity zone present in *Chlamydia* genomes are syntenic in the genomes of *Clavichlamydia* and *Chlamydiifrater*, albeit important genes like the MAC/perforin, phospholipase D (or cytotoxin in *Clavichlamydia*) were not detected. Gray connections reflect percent amino acid identity between proteins of two species.

Genomes of *Chlamydia* species are generally highly similar and contain only a few regions with a higher degree of variation. One of these variable regions is the plasticity zone (PZ), which ranges in different *Chlamydia* species from ∼5,500 to ∼55,500 bp including between six and 49 genes (Hölzer et al. 2020). The PZ is located between the genes encoding subunits of the acetyl-CoA carboxylase (*accBC*) and the purine biosynthesis genes *guaAB*, but the latter genes are absent in some chlamydial PZs (Nunes and Gomes 2014; Hölzer et al. 2020). The gene content within the PZ differs drastically between species but can contain important genes implicated in virulence and possibly tissue tropism, including a cytotoxin, phospholipase D, membrane attack complex component (MAC)/perforin, and a tryptophan biosynthesis gene cluster (Nunes and Gomes 2014) (fig. 4).

So far, the PZ has only been described in genomes of *Chlamydia* species. However, with the availability of genomes of members of two novel genera within Chlamydiaceae and Sororchlamydiaceae, we were able to check for its presence also in these genomes. While the *accBC* genes are present in members of Sororchlamydiaceae, we could not detect any other genes specific for the *Chlamydia* PZs and only a partial conservation of gene synteny over a large 200 kb genomic region (contig 1 nucleotides 110800–306700; fig. 4). In the clavichlamydial genome none of the cytotoxin, MAC/perforin, or tryptophan synthesis genes were detected, but there is some synteny with other (housekeeping) genes in *Chlamydiifrater* and the *C. trachomatis* PZ including genes right up- and downstream of the virulence-associated PZ genes. In comparison to Sororchlamydiaceae, the respective genomic region is condensed (fig. 4). A copy of the phospholipase D gene outside the PZ, which is found in all chlamydial genomes, is encoded elsewhere in the clavichlamydial genome and seems to have been duplicated (CLAVI_RS02740, CLAVI_RS02745, 34.9% aa identity) (Dimond and Hefty 2021). In *Chlamydiifrater*, the cytotoxin CT_166 is present in the respective region, which also shows more synteny including some rearrangements to the *C. trachomatis* PZ (fig. 4). Together, this suggests that a genomic region resembling the extant PZ was present in the last common ancestor of Chlamydiaceae and has diverged in members of the different genera. It is more similar in members of the sister genera *Chlamydiifrater* and *Chlamydia,* suggesting a stepwise evolution of the chlamydial PZ in the course of the adaptation to life inside fish, bird, reptile, and mammalian hosts and tissues.

### Genomic Adaptations to Fish Hosts

*Candidatus Cl. salmonicola* encodes 175 singleton genes and 25 larger gene families (with up to 54 members) that have no or only weak homologies to any known genes. Some genes in these large clavichlamydial gene families harbor motifs similar to those present in transport-associated genes, and in 14 of these OGs, 33–100% of the genes contain predicted transmembrane helices, together suggesting functions in transport, adhesion, or cell integrity. The remaining eight larger clavichlamydial families show no homology to any known motifs or protein domains. Examining the function of genes belonging to these conspicuous gene families might help to better understand how *Cl. salmonicola* cell biology and host interaction differs from other chlamydiae. The general pattern of expanded gene families, though, has been observed previously in other chlamydial species and might increase the potential for host interaction and host range (Domman et al. 2014).

Since the only known fish-pathogenic chlamydiae with available genome sequences are members of the Parilichlamydiaceae and *Cl. salmonicola*, we were interested in genes shared exclusively by members of these two families. To our surprise, we were not able to detect any genes exclusively shared between genomes of Parilichlamydiaceae and *Cl. salmonicola*. Since all genomes available for members of those two families are draft genomes, shared genes might either have been missed in our analysis, or the genetic basis of the interaction with fish hosts is indeed different. The genomes of Parilichlamydiaceae are the smallest chlamydial genomes known to date and underwent massive loss of metabolic potential (Taylor-Brown et al. 2018). Interestingly, we found some congruence in genes and pathways absent in Parilichlamydiaceae and the *Cl. salmonicola* genomes. Both groups lack a number of genes for cell division, lipopolysaccharide, and glycerolipid biosynthesis, suggesting that mechanisms for cell division and cell wall generation might differ in these fish pathogens from other chlamydiae (fig. 2) (Pillonel et al. 2018; Taylor-Brown et al. 2018). In addition, genes for heme, riboflavin, methylene tetrahydrofolate biosynthesis, the shikimate pathway, and the tricarboxylic acid cycle are missing in genomes from both groups (Taylor-Brown et al. 2018). As these pathways include a large number of genes, often distributed throughout the genome, it seems unlikely that their lack is purely due to their absence in the assemblies of the (high-quality) draft genomes of Parilichlamydiaceae and *Cl. salmonicola*. While complete genome sequences for these species would be needed to verify these observations, current evidence suggests a common pattern regarding the absence of certain metabolic traits between chlamydiae infecting fish. As the phylogenetic placement of Parilichlamydiaceae is not fully resolved yet (Dharamshi et al. 2023), it remains to be seen whether the potential loss of these genes is due to convergent evolution in a similar vertebrate host as suggested earlier (Taylor-Brown et al. 2018), or if these organisms share a closer relationship than previously assumed. In both scenarios, the lack of important metabolic traits might be the reason for the restriction of these chlamydiae to fish hosts. To gain more insights into these important questions, successful isolation and cultivation of these chlamydiae would be of great importance.

The genome sequence of *Cl. salmonicola* represents the first available genome sequence of the most deeply branching genus within the Chlamydiaceae. By comparative analysis including other recently published close relatives (Vorimore et al. 2021; Dharamshi et al. 2022), we were able to identify a set of genes that seem to play a role in the adaptation of chlamydiae from less complex metazoan hosts to mammals. Starting from sponge-associated Sorochlamydiaceae to fish and bird infecting *Cl. salmonicola* and *Chlamydiifrater* species, to bird, reptile, and mammalian *Chlamydia* pathogens, we observed evidence for stepwise adaptations in the organization of the PZ, virulence gene content and presence of protein domains of key virulence-associated genes. The genome sequence of *Cl. salmonicola* thus constitutes an important puzzle piece in our understanding of chlamydial biology and evolution.

## Materials and Methods

### Sample Preparation

Twenty Atlantic salmons (*Salmo salar*) were randomly sampled by veterinarians from a commercial fish farm in the northwest of Ireland in February 2009 (Mitchell et al. 2010). Fish were held in fresh water tanks supplied with river water. All fish were from the same cohort and had a body weight of 40–60 g. The fish showed no clinical abnormalities or pathological symptoms.

### DNA Extraction and Quality Control

Genomic DNA was isolated from five gill arches of eight individuals respectively. Gills were homogenized in a Dounce tissue grinder (Wheaton) in buffer A (35 mM Tris-HCl, 25 mM KCl, 10 mM MgCl_2_, 250 mM sucrose (all Sigma Aldrich), pH 7.5) containing 250 mM EDTA (Carl Roth). The suspension was filtered through a 5 µm syringe filter and centrifuged for 10 min at 6,000 rpm and 4 °C. The pellet was washed twice in buffer A, resuspended in buffer A containing 10 U DNase I (Thermofisher Scientific), and incubated for 1 h at 37 °C. DNase I digestion was stopped with 50 mM EDTA and centrifuged as above. The resulting pellet was washed in buffer A with 250 mM EDTA and resuspended in TE buffer (Thermofisher Scientific). Bacterial DNA was purified using a sodium dodecyl sulphate-based method including 1% hexadecylmethylammonium bromide (CTAB, Sigma Aldrich) and 200 µg/ml Proteinase K (Sigma Aldrich) in the extraction buffer (Zhou et al. 1996). Genomic DNA was stored at −20 °C until further use.

Semiquantitative PCR assays were performed to estimate the relative abundance of rRNA genes in the extracted genomic DNA. All PCR reactions were performed with 1 µl template DNA, 1 unit of Taq DNA polymerase, 10 x Taq buffer with KCl and 2 mM MgCl_2_, 0.2 mM of each deoxynucleotide (all Thermofisher Scientific) and 50 pmol of each primer in a total volume of 50 µl. Negative (without template) and positive controls in defined declining template concentrations were included in all semiquantitative PCR assays. The presence and size of amplicons were checked by gel electrophoresis and ethidium bromide or SYBR Green I (Sigma Aldrich) staining. Only low amounts (<10 pg/µl) of eukaryotic DNA were detected by the general 18S rRNA gene primer pair 18SF/18SR (supplementary table S5, Supplementary Material online). The Chlamydiae-specific primer pair SigF2/SigR2 (supplementary table S5, Supplementary Material online) yielded a strong band, which was after purification with the QIAquick PCR purification kit (Qiagen) directly sequenced (Microsynth, Austria) and had 100% identity to published clavichlamydial 16S rRNA gene sequences. The successful direct sequencing of the PCR product confirmed that no other chlamydial species had been present in the gill tissue. Finally, the use of the general bacterial primer pair 616 V/1492R (not targeting chlamydiae; supplementary table S5, Supplementary Material online) did result in a weak band only (<10 pg/µl), suggesting only traces of other bacterial DNA besides the clavichlamydial DNA in the sample.

### Genome Sequencing and Comparative Genome Analysis

Library preparation and sequencing of genomic DNA were performed at Agowa GmbH (Berlin, Germany) using a 454 GS-FLX Titanium pyrosequencing platform and at the Vienna BioCenter Core Facilities Next-Generation Sequencing Unit (https://www.viennabiocenter.org/facilities/) using an Illumina HiSeq2000 instrument to generate paired-end reads of ∼125 bases according to standard procedures. After quality filtering with BBDuk (minlen = 50 qtrim = rl trimq = 25 ktrim = r k = 25 mink = 11) from the BBTools package (v34.24, Bushnell), the reads were used for a hybrid assembly with SPAdes v3.6.0 (Bankevich et al. 2012). The assembly was screened for completeness and contamination with CheckM2 v0.1.3 (Chklovski et al. 2022). Genome annotation was performed with ConsPred 1.22 (Weinmaier et al. 2016). Comparative genome analysis was performed using 84 high-quality genomes (>90% completeness, < 5% contamination) from chlamydiae and 14 members of the Planctomycetes, Verrucomicrobia, and Lentisphaerae (supplementary table S2, Supplementary Material online). Despite a lower level of completeness and high contamination (74.31% and 33.66%, respectively), the “*Candidatus* Similichlamydia epinepheli” genome was included in the dataset to increase the number of representatives of the Parilichlamydiaceae. ANI between the genome of *Cl. salmonicola* and those of Sororchlamydiaceae, *Chlamydiifrater* and *Chlamydia* species were determined by pariwise ANIb calculation on JSpeciesWS (Richter et al. 2016) (supplementary table S2, Supplementary Material online). Average AAI between all proteome sequences in the dataset were calculated with the AAI matrix calculator in the enveomics collection (Rodriguez-R and Konstantinidis 2016), but only AAI of *Cl. salmonicola* to the respective proteome are listed in supplementary table S2, Supplementary Material online.

All proteins encoded in these genomes were clustered into OGs with OrthoFinder 2.5.4 with default parameters (Emms and Kelly 2019). For the final analysis, 49 chlamydial genomes representing well-known chlamydial families were included (supplementary table S2, Supplementary Material online). A gene was considered as being present in a chlamydial family-level lineage, when more than 50% of the genomes of this family included in the analysis encoded the gene. The presence of genes in chlamydial genomes was visualized with the R package UpSetR 1.4.0 (Conway et al. 2017). Gene synteny in the chlamydial PZ and plasmids were visualized with the R package genoPlotR 0.8.11 (Guy et al. 2010).

The analysis of protein domains was performed by combining the comparison of ClustalW sequence alignments and InterProScan 5.62–94.0 screens at EMBL-EBI (Madeira et al. 2022).

### Phylogenomic and Phylogenetic Analysis

The protein sequences of 43 conserved checkM single copy marker proteins were extracted and aligned in CheckM v1.2.2 with the “tree” workflow (supplementary table S3, Supplementary Material online) (Parks et al. 2015). We performed model testing and maximum likelihood phylogenies with IQ-TREE 2.2.2.3 (Nguyen et al. 2015) under the empirical LG model (Le and Gascuel 2008). The optimal model was determined with “-m TESTNEW” (Kalyaanamoorthy et al. 2017), including the empirical mixture models C10–C60 with the “-madd” option (best model: LG+C60+G+F) (Quang et al. 2008). We inferred 1,000 ultrafast bootstrap replicates (Hoang et al. 2017) with the “-bnni” option for bootstrap tree optimization and 1,000 replicates of the SH-like approximate likelihood ratio test (Guindon et al. 2010). The initial species tree was then used as a guide tree for posterior mean site frequency (PMSF) modeling (Wang et al. 2018) under the LG+C60+G+F model, and 100 nonparametric bootstraps were inferred. Fourteen Planctomycetota/Verrucomicrobiota species served as the outgroup for rooting the phylogenetic trees (supplementary table S2, Supplementary Material online).

As no other genome sequences are currently available for *Cl. salmonicola*, we performed additional phylogenetic tree inference using 16S rRNA gene sequences to analyze the affiliation of the genome sequence derived in this study with previously published *Cl. salmonicola* sequences (Karlsen et al. 2008; Guevara Soto et al. 2016a). We used the dataset described in Köstlbacher et al. (2021b) and added three additional clavichlamydial, two *Chlamydiifrater*, and one Sororchlamydiaceae 16S rRNA gene sequences (Karlsen et al. 2008; Guevara Soto et al. 2016a; Vorimore et al. 2021; Dharamshi et al. 2022). The sequences were aligned with SINA (Pruesse et al. 2012) and trimmed with trimAl “-gappyout” (Capella-Gutiérrez et al. 2009). After performing model finding with IQ-TREE 2.2.3 (Kalyaanamoorthy et al. 2017), the phylogenetic tree was inferred with the best-fitting model SYM+R10 and 100 nonparametric bootstraps.

In order to check for the presence of homologs of chlamydial virulence genes in other organisms, the dataset published in Dharamshi et al. (2023) was used. In addition, genes of interest were checked for homologs against OmaGroups in the Omabrowser (Altenhoff et al. 2021) (supplementary table S4, Supplementary Material online).

The phylogeny of TarP was inferred using representative sequences from the Chlamydiaceae and the respective OmaGroup (697213). Sequences were aligned with MAFFT v7.520 –auto (Katoh and Standley 2013) and a maximum likelihood phylogeny was inferred with 1,000 ultrafast bootstraps, bootstrap optimization, and 1,000 replicates of the SH-like approximate likelihood ratio test in IQ-TREE 2.2.2.3 (IQ-TREE -TESTNEW -bnni -bb 1000 -alrt 1000) (Thi Guindon et al. 2010; Nguyen et al. 2015; Hoang et al. 2017). The best model PMB+F+G4 was used for tree inference.

All phylogenetic trees generated in this study were visualized with iTOL version 6.7.5 (Letunic and Bork 2021).

## Supplementary Material

evad147_Supplementary_Data

## Data Availability

The genome sequence of *Clavichlamydia salmonicola* ET is available at DDBJ/ENA/GenBank under the accession number WTCQ00000000. The NCBI BioProject and BioSample numbers are PRJNA492195 and SAMN10090347, respectively.

## References

[evad147-B1] Altenhoff AM , et al 2021. OMA Orthology in 2021: website overhaul, conserved isoforms, ancestral gene order and more. Nucleic Acids Res. 49:D373–D379. 33174605 10.1093/nar/gkaa1007 PMC7779010

[evad147-B2] Bankevich A , et al 2012. SPAdes: a new genome assembly algorithm and its applications to single-cell sequencing. J Comput Biol. 19:455–477. 22506599 10.1089/cmb.2012.0021 PMC3342519

[evad147-B3] Blandford MI , Taylor-Brown A, Schlacher TA, Nowak B, Polkinghorne A. 2018. Epitheliocystis in fish: an emerging aquaculture disease with a global impact. Transbound Emerg Dis. 65:1436–1446. 29790651 10.1111/tbed.12908

[evad147-B4] Borel N , Polkinghorne A, Pospischil A. 2018. Chlamydial diseases in animals: still a challenge for pathologists? Vet Pathol. 55:374–390. 29310550 10.1177/0300985817751218

[evad147-B5] Bradley T , Newcomer C, Maxwell K. 1988. Epitheliocystis associated with massive mortalities of cultured lake trout *Saivelinus namaycush*. Dis Aquat Organ. 4:9–17.

[evad147-B6] Braun C , et al 2019. CPN0572, the *C. pneumoniae* ortholog of TarP, reorganizes the actin cytoskeleton via a newly identified F-actin binding domain and recruitment of vinculin. PLoS One. 14:e0210403. 30629647 10.1371/journal.pone.0210403 PMC6328165

[evad147-B7] Bugalhão JN , Mota LJ. 2019. The multiple functions of the numerous *Chlamydia trachomatis* secreted proteins: the tip of the iceberg. Microb Cell. 6:414–449. 31528632 10.15698/mic2019.09.691 PMC6717882

[evad147-B8] Bulir DC , et al 2014. *Chlamydia pneumoniae* CopD translocator protein plays a critical role in type III secretion (T3S) and infection. PLoS One. 9:e99315. 24959658 10.1371/journal.pone.0099315 PMC4068993

[evad147-B9] Bushnell B . BBtools. sourceforge.net/projects/bbmap/.

[evad147-B10] Capella-Gutiérrez S , Silla-Martínez JM, Gabaldón T. 2009. Trimal: a tool for automated alignment trimming in large-scale phylogenetic analyses. Bioinformatics 25:1972–1973. 19505945 10.1093/bioinformatics/btp348 PMC2712344

[evad147-B11] Caven L , Carabeo RA. 2020. Pathogenic puppetry: manipulation of the host actin cytoskeleton by *Chlamydia trachomatis*. Int J Mol Sci. 21:90. 10.3390/ijms21010090 PMC6981773 31877733

[evad147-B12] Chamberlain NB , Dimond Z, Hackstadt T. 2022. *Chlamydia trachomatis* suppresses host cell store-operated Ca2 + entry and inhibits NFAT/calcineurin signaling. Sci Rep. 12: 21406. 36496532 10.1038/s41598-022-25786-y PMC9741641

[evad147-B13] Chklovski A , Parks DH, Woodcroft BJ, Tyson GW. 2022. CheckM2: a rapid, scalable and accurate tool for assessing microbial genome quality using machine learning. bioRxiv. doi:10.1101/2022.07.11.499243, preprint: not peer reviewed. 37500759

[evad147-B14] Collingro A , Köstlbacher S, Horn M. 2020. Chlamydiae in the environment. Trends Microbiol. 28:877–888. 32591108 10.1016/j.tim.2020.05.020

[evad147-B15] Conway JR , Lex A, Gehlenborg N. 2017. Upsetr: an R package for the visualization of intersecting sets and their properties. Bioinformatics 33:2938–2940. 28645171 10.1093/bioinformatics/btx364 PMC5870712

[evad147-B16] Dharamshi JE , et al 2020. Marine sediments illuminate chlamydiae diversity and evolution. Curr Biol. 30:1032–1048.e7. 32142706 10.1016/j.cub.2020.02.016

[evad147-B17] Dharamshi JE , et al 2022. Genomic diversity and biosynthetic capabilities of sponge-associated chlamydiae. ISME J. 16:2725–2740. 36042324 10.1038/s41396-022-01305-9 PMC9666466

[evad147-B18] Dharamshi JE , et al 2023. Gene gain facilitated endosymbiotic evolution of Chlamydiae. Nat Microbiol. 8:40–54. 36604515 10.1038/s41564-022-01284-9 PMC9816063

[evad147-B19] Dimond ZE , Hefty PS. 2021. Comprehensive genome analysis and comparisons of the swine pathogen, *Chlamydia suis* reveals unique ORFs and candidate host-specificity factors. Pathog Dis. 79:ftaa035. 32639528 10.1093/femspd/ftaa035 PMC7948067

[evad147-B20] Domman D , et al 2014. Massive expansion of ubiquitination-related gene families within the Chlamydiae. Mol Biol Evol. 31:2890–2904. 25069652 10.1093/molbev/msu227 PMC4209131

[evad147-B21] Domman D , Horn M. 2015. Following the footsteps of chlamydial gene regulation. Mol Biol Evol. 32:3035–3046. 26424812 10.1093/molbev/msv193 PMC4652624

[evad147-B22] Elwell C , Mirrashidi K, Engel J. 2016. Chlamydia cell biology and pathogenesis. Nat Rev Microbiol. 14:385–400. 27108705 10.1038/nrmicro.2016.30 PMC4886739

[evad147-B23] Emms DM , Kelly S. 2019. Orthofinder: phylogenetic orthology inference for comparative genomics. Genome Biol. 20:238. 31727128 10.1186/s13059-019-1832-y PMC6857279

[evad147-B24] Ende RJ , Murray RL, D'spain SK, Coppens I, Derré I. 2022. Phosphoregulation accommodates type III secretion and assembly of a tether of ER-*Chlamydia* inclusion membrane contact sites. Elife 11:e74535. 35838228 10.7554/eLife.74535 PMC9286742

[evad147-B25] Ghosh S et al 2020. Fluorescence-reported allelic exchange mutagenesis-mediated gene deletion indicates a requirement for *Chlamydia trachomatis* TarP during in vivo infectivity and reveals a specific role for the C terminus during cellular invasion. https://journals.asm.org/journal/iai. 10.1128/IAI.00841-19 PMC7171248 32152196

[evad147-B26] Gitsels A , Sanders N, Vanrompay D. 2019. Chlamydial infection from outside to inside. Front Microbiol. 10:2329. 31649655 10.3389/fmicb.2019.02329 PMC6795091

[evad147-B27] Guevara Soto M , et al 2016a. Epitheliocystis distribution and characterization in brown trout (*Salmo trutta*) from the headwaters of two major European rivers, the Rhine and Rhone. Front Physiol. 7:131. 27148070 10.3389/fphys.2016.00131 PMC4834352

[evad147-B28] Guevara Soto M , et al 2016b. The emergence of epitheliocystis in the upper Rhone region: evidence for Chlamydiae in wild and farmed salmonid populations. Arch Microbiol. 198:315–324. 26802008 10.1007/s00203-016-1192-x

[evad147-B29] Guevara Soto M , et al 2017. Investigations into the temporal development of epitheliocystis infections in brown trout: a histological study. J Fish Dis. 40:811–819. 27670837 10.1111/jfd.12562

[evad147-B30] Guindon S , et al 2010. New algorithms and methods to estimate maximum-likelihood phylogenies: assessing the performance of PhyML 3.0. Syst Biol. 59:307–321. 20525638 10.1093/sysbio/syq010

[evad147-B31] Guy L , Roat Kultima J, Andersson SGE. 2010. Genoplotr: comparative gene and genome visualization in R. Bioinformatics 26:2334–2335. 20624783 10.1093/bioinformatics/btq413 PMC2935412

[evad147-B32] Hamaoui D et al 2020. The Chlamydia effector CT622/TaiP targets a nonautophagy related function of ATG16L1. Proc Natl Acad Sci U S A. 2020;117:26784–26794.. 33055216 10.1073/pnas.2005389117 PMC7604492

[evad147-B33] Hoang DT , Chernomor O, von Haeseler A, Minh BQ, Vinh LS. 2017. UFBoot2: improving the ultrafast bootstrap approximation. Mol Biol Evol. 35:518–522. 10.1093/molbev/msx281 PMC5850222 29077904

[evad147-B34] Hölzer M , et al 2020. Comparative genome analysis of 33 chlamydia strains reveals characteristic features of *Chlamydia psittaci* and closely related species. Pathogens 9:899. 33126635 10.3390/pathogens9110899 PMC7694038

[evad147-B35] Jewett TJ , Fischer ER, Mead DJ, Hackstadt T. 2006. Chlamydial TarP is a bacterial nucleator of actin. Proc Natl Acad Sci U S A. 103:15599–15604. 17028176 10.1073/pnas.0603044103 PMC1622868

[evad147-B36] Jewett TJ , Miller NJ, Dooley CA, Hackstadt T. 2010. The conserved tarp actin binding domain is important for chlamydial invasion. PLoS Pathog. 6: e1000997. 20657821 10.1371/journal.ppat.1000997 PMC2904776

[evad147-B37] Kalyaanamoorthy S , Minh BQ, Wong TKF, Von Haeseler A, Jermiin LS. 2017. Modelfinder: fast model selection for accurate phylogenetic estimates. Nat Methods. 14:587–589. 28481363 10.1038/nmeth.4285 PMC5453245

[evad147-B38] Karlsen M , et al 2008. Characterization of ‘*Candidatus* clavochlamydia salmonicola’: an intracellular bacterium infecting salmonid fish. Environ Microbiol. 10:208–218. 17894816 10.1111/j.1462-2920.2007.01445.x

[evad147-B39] Katharios P , et al 2015. Environmental marine pathogen isolation using mesocosm culture of sharpsnout seabream: striking genomic and morphological features of novel *Endozoicomonas* sp. Sci Rep. 5:17609. 26639610 10.1038/srep17609 PMC4671022

[evad147-B40] Katoh K , Standley DM. 2013. MAFFT Multiple sequence alignment software version 7: improvements in performance and usability. Mol Biol Evol. 30:772–780. 23329690 10.1093/molbev/mst010 PMC3603318

[evad147-B41] Konstantinidis KT , Rosselló-Móra R, Amann R. 2017. Uncultivated microbes in need of their own taxonomy. ISME J. 11:2399–2406. 28731467 10.1038/ismej.2017.113 PMC5649169

[evad147-B42] Köstlbacher S , et al 2021b. Pangenomics reveals alternative environmental lifestyles among chlamydiae. Nat Commun. 12:4021. 34188040 10.1038/s41467-021-24294-3 PMC8242063

[evad147-B43] Köstlbacher S , Collingro A, Halter T, Domman D, Horn M. 2021a. Coevolving plasmids drive gene flow and genome plasticity in host-associated intracellular bacteria. Curr Biol. 31:346–357.e3. 33157023 10.1016/j.cub.2020.10.030 PMC7846284

[evad147-B44] Le SQ , Gascuel O. 2008. An improved general amino acid replacement matrix. Mol Biol Evol. 25:1307–1320. 18367465 10.1093/molbev/msn067

[evad147-B45] Lei L , et al 2021. A chlamydial plasmid-dependent secretion system for the delivery of virulence factors to the host cytosol. mBio 12:e0117921. 34101486 10.1128/mBio.01179-21 PMC8262877

[evad147-B46] Letunic I , Bork P. 2021. Interactive tree of life (iTOL) v5: an online tool for phylogenetic tree display and annotation. Nucleic Acids Res. 49:W293–W296. 33885785 10.1093/nar/gkab301 PMC8265157

[evad147-B47] Lorenzini E , et al 2010. Structure and protein-protein interaction studies on *Chlamydia trachomatis* protein CT670 (YscO homolog). J Bacteriol. 192:2746–2756. 20348249 10.1128/JB.01479-09 PMC2876502

[evad147-B48] Lutter EI , et al 2010. Phylogenetic analysis of *Chlamydia trachomatis* TarP and correlation with clinical phenotype. Infect Immun. 78:3678–3688. 20605986 10.1128/IAI.00515-10 PMC2937449

[evad147-B49] Madeira F , et al 2022. Search and sequence analysis tools services from EMBL-EBI in 2022. Nucleic Acids Res. 50:W276–W279. 35412617 10.1093/nar/gkac240 PMC9252731

[evad147-B50] Matteï PJ , et al 2011. Membrane targeting and pore formation by the type III secretion system translocon. FEBS J. 278:414–426. 21182592 10.1111/j.1742-4658.2010.07974.x

[evad147-B51] Mitchell SO , et al 2010. Epitheliocystis in Atlantic salmon, *Salmo salar* L., farmed in fresh water in Ireland is associated with ‘*Candidatus* Clavochlamydia salmonicola’ infection. J Fish Dis. 33:665–673. 20629856 10.1111/j.1365-2761.2010.01171.x

[evad147-B52] Mueller KE , Plano G V, Fields KA. 2014. New frontiers in type III secretion biology: the Chlamydia perspective. Infect Immun. 82:2–9. 24126521 10.1128/IAI.00917-13 PMC3911841

[evad147-B53] Nguyen PH , Lutter EI, Hackstadt T. 2018. Chlamydia trachomatis inclusion membrane protein MrcA interacts with the inositol 1,4,5-trisphosphate receptor type 3 (ITPR3) to regulate extrusion formation. PLoS Pathog. 14:e1006911. 29543918 10.1371/journal.ppat.1006911 PMC5854415

[evad147-B54] Nguyen L-T , Schmidt HA, von Haeseler A, Minh BQ. 2015. IQ-TREE: a fast and effective stochastic algorithm for estimating maximum-likelihood phylogenies. Mol Biol Evol. 32:268–274. 25371430 10.1093/molbev/msu300 PMC4271533

[evad147-B55] Nunes A , Gomes JP. 2014. Evolution, phylogeny, and molecular epidemiology of *Chlamydia*. Infection. Genet Evol. 23:49–64. 10.1016/j.meegid.2014.01.029 24509351

[evad147-B56] Parks DH , Imelfort M, Skennerton CT, Hugenholtz P, Tyson GW. 2015. Checkm: assessing the quality of microbial genomes recovered from isolates, single cells, and metagenomes. Genome Res. 25:1043–1055. 25977477 10.1101/gr.186072.114 PMC4484387

[evad147-B57] Parrett CJ , Lenoci R V, Nguyen B, Russell L, Jewett TJ. 2016. Targeted disruption of *Chlamydia trachomatis* invasion by in trans expression of dominant negative tarp effectors. Front Cell Infect Microbiol. 6:84. 27602332 10.3389/fcimb.2016.00084 PMC4993794

[evad147-B58] Pawlikowska-Warych M , Deptuła W. 2016. Characteristics of chlamydia-like organisms pathogenic to fish. J Appl Genet. 57:135–141. 26160214 10.1007/s13353-015-0303-8 PMC4731428

[evad147-B59] Pedrosa AT , et al 2020. A post-invasion role for chlamydia type III effector TarP in modulating the dynamics and organization of host cell focal adhesions. J Biol Chemistry. 295:14763–14779. 10.1074/jbc.RA120.015219 PMC7586217 32843479

[evad147-B60] Peters J , Wilson DP, Myers G, Timms P, Bavoil PM. 2007. Type III secretion à la Chlamydia. Trends Microbiol. 15:241–251. 17482820 10.1016/j.tim.2007.04.005

[evad147-B61] Pillonel T , Bertelli C, Greub G. 2018. Environmental metagenomic assemblies reveal seven new highly divergent chlamydial lineages and hallmarks of a conserved intracellular lifestyle. Front Microbiol. 9:79. 29515524 10.3389/fmicb.2018.00079 PMC5826181

[evad147-B62] Pruesse E , Peplies J, Glöckner FO. 2012. SINA: accurate high-throughput multiple sequence alignment of ribosomal RNA genes. Bioinformatics 28:1823–1829. 22556368 10.1093/bioinformatics/bts252 PMC3389763

[evad147-B63] Quang LS , Gascuel O, Lartillot N. 2008. Empirical profile mixture models for phylogenetic reconstruction. Bioinformatics 24:2317–2323. 18718941 10.1093/bioinformatics/btn445

[evad147-B64] Richter M , Rosselló-Móra R, Oliver Glöckner F, Peplies J. 2016. JSpeciesWS: a web server for prokaryotic species circumscription based on pairwise genome comparison. Bioinformatics 32:929–931. 26576653 10.1093/bioinformatics/btv681 PMC5939971

[evad147-B65] Rodriguez-R LM , Konstantinidis KT. 2016. The enveomics collection: a toolbox for specialized analyses of microbial genomes and metagenomes. PeerJ Preprints 4:e1900v1.

[evad147-B66] Rourke AW , Davis RW, Bradley TM. 1984. A light and electron microscope study of epitheliocystis in juvenile steelhead trout, *Salmo gairdneri* Richardson. J Fish Dis. 7:301–309.

[evad147-B67] Schmidt-Posthaus H , et al 2012. A natural freshwater origin for two chlamydial species, *Candidatus* piscichlamydia salmonis and *Candidatus* Clavochlamydia salmonicola, causing mixed infections in wild brown trout (*Salmo trutta*). Environ Microbiol. 14:2048–2057. 22176683 10.1111/j.1462-2920.2011.02670.x

[evad147-B68] Seth-Smith HMB , et al 2016. Emerging pathogens of gilthead seabream: characterisation and genomic analysis of novel intracellular β-proteobacteria. ISME J. 10:1791–1803. 26849311 10.1038/ismej.2015.223 PMC4861247

[evad147-B69] Shehat MG , Aranjuez GF, Kim J, Jewett TJ. 2021. The *Chlamydia trachomatis* TarP effector targets the Hippo pathway. Biochem Biophys Res Commun. 562:133–138. 34052658 10.1016/j.bbrc.2021.05.057 PMC8206025

[evad147-B70] Stride MC , Polkinghorne A, Nowak BF. 2014. Chlamydial infections of fish: diverse pathogens and emerging causes of disease in aquaculture species. Vet Microbiol. 171:258–266. 24932463 10.1016/j.vetmic.2014.03.022

[evad147-B71] Taylor-Brown A , et al 2017. Culture-independent genomics of a novel chlamydial pathogen of fish provides new insight into host-specific adaptations utilized by these intracellular bacteria. Environ Microbiol. 19:1899–1913. 28205377 10.1111/1462-2920.13694

[evad147-B72] Taylor-Brown A , et al 2018. Metagenomic analysis of fish-associated Ca. Parilichlamydiaceae reveals striking metabolic similarities to the terrestrial Chlamydiaceae. Genome Biol Evol. 10:2614–2628. 30202970 10.1093/gbe/evy195 PMC6171736

[evad147-B73] Taylor-Brown A , Vaughan L, Greub G, Timms P, Polkinghorne A. 2015. Twenty years of research into *Chlamydia*-like organisms: a revolution in our understanding of the biology and pathogenicity of members of the phylum Chlamydiae. Pathog Dis. 73:1–15. 10.1093/femspd/ftu009 25854000

[evad147-B74] Thwaites T , et al 2014. The *Chlamydia* effector TarP mimics the mammalian leucine-aspartic acid motif of paxillin to subvert the focal adhesion kinase during invasion. J Biol Chem. 289:30426–30442. 25193659 10.1074/jbc.M114.604876 PMC4215226

[evad147-B75] Thwaites TR , Pedrosa AT, Peacock TP, Carabeo RA. 2015. Vinculin interacts with the *Chlamydia* effector tarP via a tripartite vinculin binding domain to mediate actin recruitment and assembly at the plasma membrane. Front Cell Infect Microbiol. 5:88. 26649283 10.3389/fcimb.2015.00088 PMC4663276

[evad147-B76] Toenshoff ER , et al 2012. A novel betaproteobacterial agent of gill epitheliocystis in seawater farmed atlantic salmon (*Salmo salar*). PLoS One. 7:e32696. 22427865 10.1371/journal.pone.0032696 PMC3299688

[evad147-B77] Vorimore F , et al 2021. Evidence for the existence of a new genus *Chlamydiifrater* gen. nov. inside the family Chlamydiaceae with two new species isolated from flamingo (*Phoenicopterus roseus*): *Chlamydiifrater phoenicopteri* sp. nov. and *Chlamydiifrater volucris* sp. nov. Syst Appl Microbiol. 44:126200. 34298369 10.1016/j.syapm.2021.126200

[evad147-B78] Vromman F , Perrinet S, Gehre L, Subtil A. 2016. The DUF582 proteins of *Chlamydia trachomatis* bind to components of the ESCRT machinery, which is dispensable for bacterial growth in vitro. Front Cell Infect Microbiol. 6:123. 27774439 10.3389/fcimb.2016.00123 PMC5053991

[evad147-B79] Wang HC , Minh BQ, Susko E, Roger AJ. 2018. Modeling site heterogeneity with posterior mean site frequency profiles accelerates accurate phylogenomic estimation. Syst Biol. 67:216–235. 28950365 10.1093/sysbio/syx068

[evad147-B80] Weinmaier T , et al 2016. Conspred: a rule-based (re-)annotation framework for prokaryotic genomes. Bioinformatics 32:3327–3329. 27378292 10.1093/bioinformatics/btw393

[evad147-B81] Yu HHY , Kibler D, Tan M. 2006. In silico prediction and functional validation of σ28-regulated genes in *Chlamydia* and *Escherichia coli*. J Bacteriol. 188:8206–8212. 16997971 10.1128/JB.01082-06 PMC1698183

[evad147-B82] Zhou J , Bruns MA, Tiedje JM. 1996. DNA recovery from soils of diverse composition. Appl Environ Microbiol. 62:316–322. 8593035 10.1128/aem.62.2.316-322.1996 PMC167800

