# Supplementary material for: The Fish Pathogen “Candidatus Clavichlamydia salmonicola”—A Missing Link in the Evolution of Chlamydial Pathogens of Humans
Source: Genome Biol Evol. 2023 Aug 24;15(8):evad147. doi: 10.1093/gbe/evad147 (PMC10448858; doi:10.1093/gbe/evad147)
Supplement: evad147_Supplementary_Data [file evad147_supplementary_data.zip › Supplementary material_revised.pdf]

The fish pathogen '*Candidatus* Clavichlamydia salmonicola' - a missing link in the evolution of chlamydial pathogens of humans

Astrid Collingro[1*], Stephan Köstlbacher[1,2,3], Alexander Siegl[1], Elena R. Toenshoff[1,4], Frederik Schulz[1,5], Susan O. Mitchell[6], Thomas Weinmaier[1], Thomas Rattei[1], Duncan J. Colquhoun[7], Matthias Horn[1]

[1] Centre for Microbiology and Environmental Systems Science, University of Vienna, Vienna, Austria.
[2] Doctoral School in Microbiology and Environmental Science, University of Vienna, Vienna, Austria
[3] Laboratory of Microbiology, Wageningen University and Research, Wageningen, The Netherlands.
[4] Institute of Molecular Biology and Biophysics, Eidgenössische Technische Hochschule Zürich (ETH), Zürich, Switzerland.
[5] DOE Joint Genome Institute, Berkeley, CA, USA.
[6] FishVet Group Ireland, Oranmore, Co. Galway, Ireland.
[7] Department of Biological Sciences, University of Bergen, Bergen, Norway.

Supplementary tables and figures

1. Supplementary tables

Supplementary table 1. General features of the *Clavichlamydia salmonicola* ET genome.

Feature	Chromosome	Plasmid
Size (bp)	1,384,950	8,040
G+C content (%)	32.5	28
Scaffolds	27	1
CDS	1,096	9
rRNA genes	3 (1 operon)	0
tRNA genes	37	0
ncRNA	3	0

Supplementary table 2. Genome sequences used for phylogenetic analysis and the generation of orthologous gene families (see separate file).

Supplementary table 3. CheckM marker genes used for phylogenetic analysis.

Protein family accession	Description	Protein family accession	Description
**PF00164**	Ribosomal protein S12/S23	PF00573	Ribosomal protein L4/L1 family
**PF00177**	Ribosomal protein S7p/S5e	PF00623	RNA polymerase Rbp1, domain 2
**PF00181**	Ribosomal protein L2, RNA binding domain	PF00673	Ribosomal L5P family C-terminus
**PF00189**	Ribosomal protein S3, C-terminal domain	PF00687	Ribosomal protein L1p/L10e family
**PF00203**	Ribosomal protein S19	PF00831	Ribosomal protein L29
**PF00237**	Ribosomal protei L22p/L17e	PF00861	Ribosomal protein L18
**PF00238**	Ribosomal protein L14p/L23e	PF01192	RNA polymerase Rpb6
**PF00252**	Ribosomal protein L16p/L10e	PF01509	TruB family pseudouridylate synthase (N-terminal domain)
**PF00276**	Ribosomal protein L23	PF02978	Signal peptide binding domain
**PF00281**	Ribosomal protein L5P family C-terminus	PF03719	Ribosomal protein S5, C-terminal domain
**PF00297**	Ribosomal protein L3	PF03946	Ribosomal protein L11, N-terminal domain
**PF00298**	Ribosomal protein L11, RNA binding domain	PF03947	Ribosomal protein L2, C-terminal domain
**PF00312**	Ribosomal protein S15	PF04560	RNA polymerase Rpb2, domain 7
**PF00318**	Ribosomal protein S2	PF04561	RNA polymerase Rpb2, domain 2
**PF00333**	Ribosomal protein S5, N-terminal domain	PF04563	RNA polymerase beta subunit
**PF00366**	Ribosomal protein S17	PF04565	RNA polymerase Rpb2, domain 3
**PF00380**	Ribosomal protein S9/S16	PF04997	RNA polymerase Rpb1, domain 1
**PF00410**	Ribosomal protein S8	PF05000	RNA polymerase Rpb1, domain 4
**PF00411**	Ribosomal protein S11	PF11987	Translation-initiation factor 2
**PF00466**	Ribosomal protein L10	TIGR00344	Alanine--tRNA ligase
**PF00562**	RNA polymerase Rpb2, domain 6	TIGR00422	Valine--tRNA ligase
**PF00572**	Ribosomal protein L13		

Supplementary table 4. Origin of genes important in chlamydial infections and specific to Sororchlamydiaceae and Chlamydiaceae (see separate file).

Supplementary table 5. Primers used in this study.

Short name	Sequence (5' – 3')	Specificity	Target site[a]	Annealing temp.	Reference
**18SF**	GTA GTC ATA TGC TTG TCT C	18S rRNA gene, Amoebozoa	17-35	52°C	Schmitz-Esser et al.
**18SR**	CGR ARA CCT TGT TAC GAC	18S rRNA gene, Amoebozoa	2256-2273	52°C	Schmitz-Esser et al.
**SigF2**	CRG CGT GGA TGA GGC AT	16S rRNA gene, *Chlamydiales*	40-56	60°C	Haider et al.
**SigR2**	TCA GTC CCA RTG TTG GC	16S rRNA gene, *Chlamydiales*	309-325	60°C	Haider et al.
**616V**	AGA GTT TGA TYM TGG CTC	16S rRNA gene, most *Bacteria*	8-25	54°C	Juretschko et al.
**1492R**	GGY TAC CTT GTT ACG ACT T	16S rRNA gene, most *Bacteria* and *Archaea*	1492-1510	54°C	Loy et al.

[a] Target site according to *E. coli* 16S rRNA or *A. castellanii* NEFF 18S rRNA gene numbering.

2. Supplementary figures

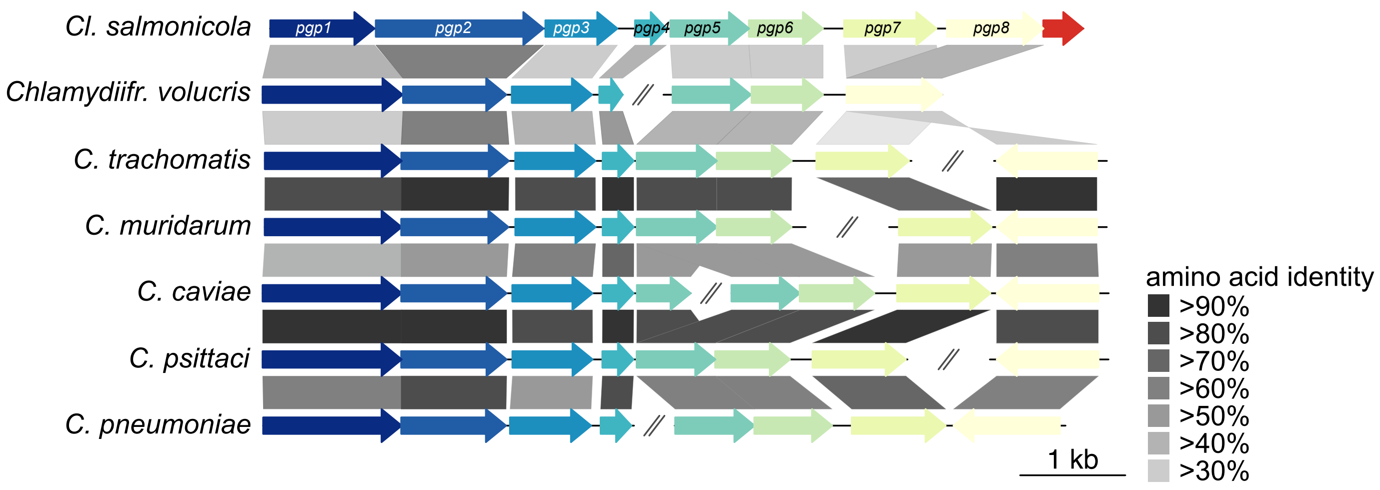

Supplementary fig. 1. Genetic organization of Chlamydiaceae plasmids. The plasmids of Chlamydiaceae members are highly similar in gene content, gene organization, and amino acid sequence identity. Plasmid genes are depicted in different colors. The red gene encodes a partial DNA helicase and is only present on the clavichlamydial plasmid. Gray connections represent percent of amino acid identity between proteins of two chlamydial species.

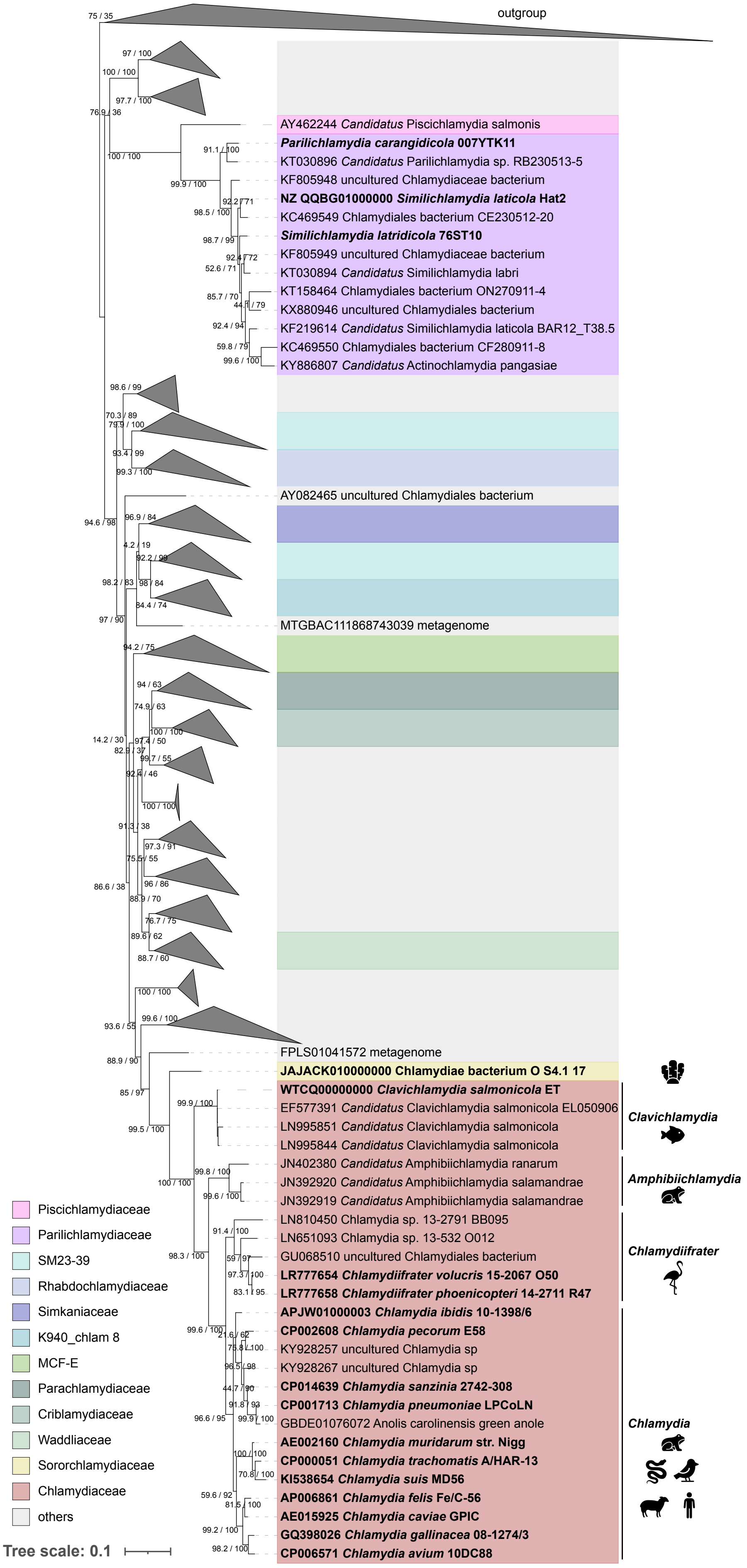
outgroup
AY462244 Candidatus Piscichlamydia salmonis
Parilichlamydia carangidicola 007YTK11
KT030896 Candidatus Parilichlamydia sp. RB230513-5
KF805948 uncultured Chlamydiaceae bacterium
NZ QQBG01000000 Similichlamydia laticola Hat2
KC469549 Chlamydiales bacterium CE230512-20
Similichlamydia latridicola 76ST10
KF805949 uncultured Chlamydiaceae bacterium
KT030894 Candidatus Similichlamydia labri
KT158464 Chlamydiales bacterium ON270911-4
KX880946 uncultured Chlamydiales bacterium
KF219614 Candidatus Similichlamydia laticola BAR12_T38.5
KC469550 Chlamydiales bacterium CF280911-8
KY886807 Candidatus Actinochlamydia pangasiae
AY082465 uncultured Chlamydiales bacterium
MTGBAC111868743039 metagenome
FPLS01041572 metagenome
JAJACK010000000 Chlamydiae bacterium O S4.1 17
WTCQ00000000 Clavichlamydia salmonicola ET
EF577391 Candidatus Clavichlamydia salmonicola EL050906
LN995851 Candidatus Clavichlamydia salmonicola
LN995844 Candidatus Clavichlamydia salmonicola
JN402380 Candidatus Amphibiichlamydia ranarum
JN392920 Candidatus Amphibiichlamydia salamandrae
JN392919 Candidatus Amphibiichlamydia salamandrae
LN810450 Chlamydia sp. 13-2791 BB095
LN651093 Chlamydia sp. 13-532 O012
GU068510 uncultured Chlamydiales bacterium
LR777654 Chlamydiifrater volucris 15-2067 O50
LR777658 Chlamydiifrater phoenicopteri 14-2711 R47
APJW01000003 Chlamydia ibidis 10-1398/6
CP002608 Chlamydia pecorum E58
KY928257 uncultured Chlamydia sp
KY928267 uncultured Chlamydia sp
CP014639 Chlamydia sanzinia 2742-308
CP001713 Chlamydia pneumoniae LPCoLN
GBDE01076072 Anolis carolinensis green anole
AE002160 Chlamydia muridarum str. Nigg
CP000051 Chlamydia trachomatis A/HAR-13
KI538654 Chlamydia suis MD56
AP006861 Chlamydia felis Fe/C-56
AE015925 Chlamydia caviae GPIC
GQ398026 Chlamydia gallinacea 08-1274/3
CP006571 Chlamydia avium 10DC88
Clavichlamydia
Amphibiichlamydia
Chlamydiifrater
Chlamydia
Piscichlamydiaceae
Parilichlamydiaceae
SM23-39
Rhabdochlamydiaceae
Simkaniaceae
K940_chlam 8
MCF-E
Parachlamydiaceae
Criblamydiaceae
Waddliaceae
Sororchlamydiaceae
Chlamydiaceae
others
Tree scale: 0.1

Supplementary fig. 2. The 16S rRNA gene encoded in the *Cl. salmonicola* genome is nearly identical to known clavichlamydial sequences, together establishing a sister genus within the Chlamydiaceae. 16S rRNA based maximum-likelihood tree inferred with IQ-TREE under the SYM+R10 model with 1000 ultrafast bootstraps and 1000 replicates of the SH-like approximate likelihood ratio test including sequences larger than 1,200 nt. The phylogenetic tree is rooted with sequences from Planctomycetes and Verrucomicrobia as an outgroup. With the exception of clavichlamydial sequences, all sequences were dereplicated at 99% identity. Chlamydial family lineages are depicted in colored ranges. Bold species were also used for genome comparison (see Fig. 1 and Supplementary table 2). Values on branches indicate ultrafast bootstrap support values and SH-like approximate likelihood ratio test support values, respectively. For Sororchlamydiaceae and Chlamydiaceae representative host organisms from the respective phylum or vertebrate class are depicted (illustrations have been downloaded from Flaticon.com).

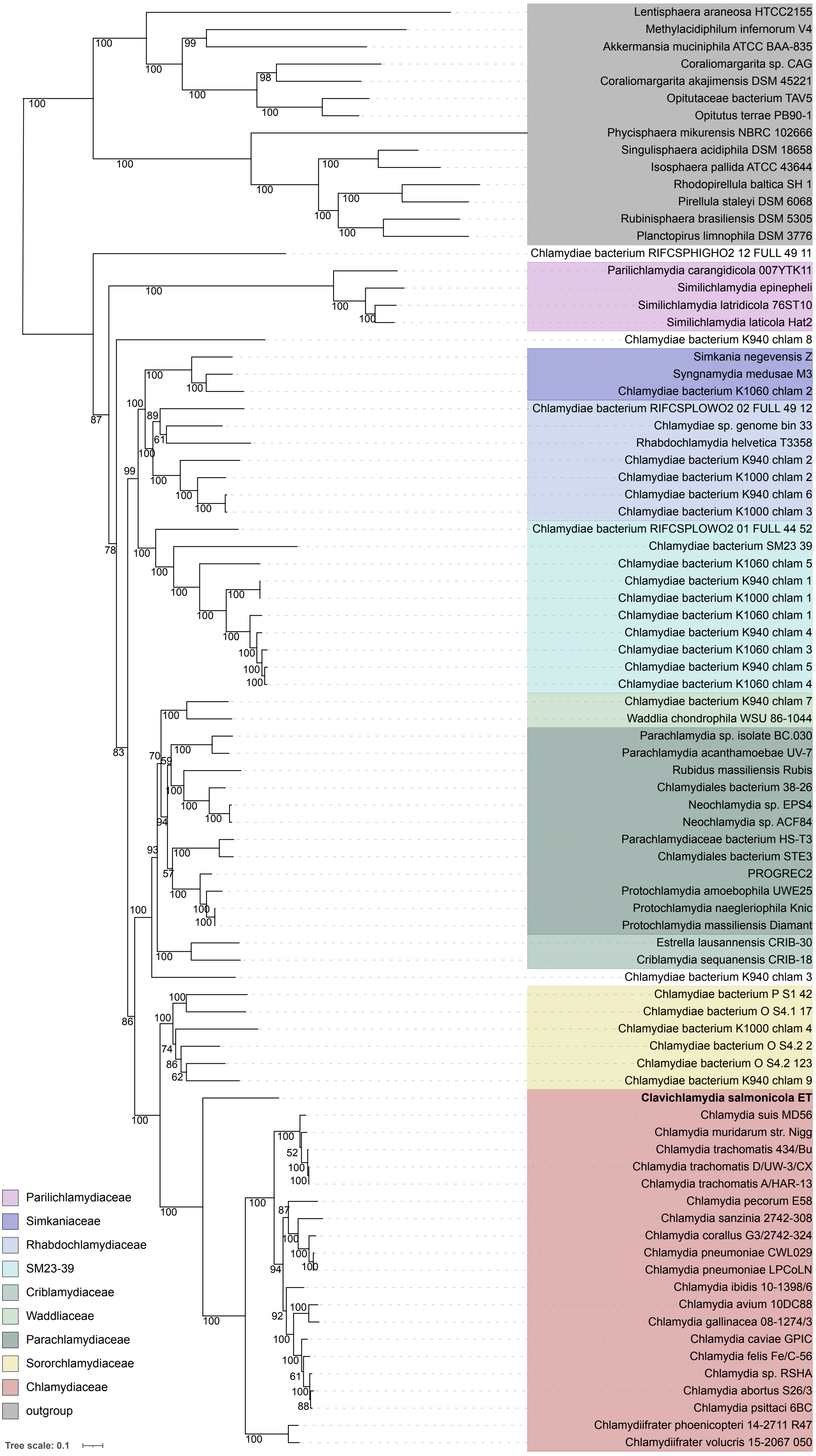

Lentisphaera araneosa HTCC2155
Methylacidiphilum infernorum V4
Akkermansia muciniphila ATCC BAA-835
Coraliomargarita sp. CAG
Coraliomargarita akajimensis DSM 45221
Opitutaceae bacterium TAV5
Opitutus terrae PB90-1
Phycisphaera mikurensis NBRC 102666
Singulisphaera acidiphila DSM 18658
Isosphaera pallida ATCC 43644
Rhodopirellula baltica SH 1
Pirellula staleyi DSM 6068
Rubinisphaera brasiliensis DSM 5305
Planctopirus limnophila DSM 3776
Chlamydiae bacterium RIFCSPHIGHO2 12 FULL 49 11
Parilichlamydia carangidicola 007YTK11
Similichlamydia epinepheli
Similichlamydia latridicola 76ST10
Similichlamydia laticola Hat2
Chlamydiae bacterium K940 chlam 8
Simkania negevensis Z
Syngnamydia medusae M3
Chlamydiae bacterium K1060 chlam 2
Chlamydiae bacterium RIFCSPLOWO2 02 FULL 49 12
Chlamydiae sp. genome bin 33
Rhabdochlamydia helvetica T3358
Chlamydiae bacterium K940 chlam 2
Chlamydiae bacterium K1000 chlam 2
Chlamydiae bacterium K940 chlam 6
Chlamydiae bacterium K1000 chlam 3
Chlamydiae bacterium RIFCSPLOWO2 01 FULL 44 52
Chlamydiae bacterium SM23 39
Chlamydiae bacterium K1060 chlam 5
Chlamydiae bacterium K940 chlam 1
Chlamydiae bacterium K1000 chlam 1
Chlamydiae bacterium K1060 chlam 1
Chlamydiae bacterium K940 chlam 4
Chlamydiae bacterium K1060 chlam 3
Chlamydiae bacterium K940 chlam 5
Chlamydiae bacterium K1060 chlam 4
Chlamydiae bacterium K940 chlam 7
Waddlia chondrophila WSU 86-1044
Parachlamydia sp. isolate BC.030
Parachlamydia acanthamoebae UV-7
Rubidus massiliensis Rubis
Chlamydiales bacterium 38-26
Neochlamydia sp. EPS4
Neochlamydia sp. ACF84
Parachlamydiaceae bacterium HS-T3
Chlamydiales bacterium STE3
PROGREC2
Protochlamydia amoebophila UWE25
Protochlamydia naegleriophila Knic
Protochlamydia massiliensis Diamant
Estrella lausannensis CRIB-30
Criblamydia sequanensis CRIB-18
Chlamydiae bacterium K940 chlam 3
Chlamydiae bacterium P S1 42
Chlamydiae bacterium O S4.1 17
Chlamydiae bacterium K1000 chlam 4
Chlamydiae bacterium O S4.2 2
Chlamydiae bacterium O S4.2 123
Chlamydiae bacterium K940 chlam 9
Clavichlamydia salmonicola ET
Chlamydia suis MD56
Chlamydia muridarum str. Nigg
Chlamydia trachomatis 434/Bu
Chlamydia trachomatis D/UW-3/CX
Chlamydia trachomatis A/HAR-13
Chlamydia pecorum E58
Chlamydia sanzinia 2742-308
Chlamydia corallus G3/2742-324
Chlamydia pneumoniae CWL029
Chlamydia pneumoniae LPCoLN
Chlamydia ibidis 10-1398/6
Chlamydia avium 10DC88
Chlamydia gallinacea 08-1274/3
Chlamydia caviae GPIC
Chlamydia felis Fe/C-56
Chlamydia sp. RSHA
Chlamydia abortus S26/3
Chlamydia psittaci 6BC
Chlamydiifrater phoenicopteri 14-2711 R47
Chlamydiifrater volucris 15-2067 050
Parilichlamydiaceae
Simkaniaceae
Rhabdochlamydiaceae
SM23-39
Criblamydiaceae
Waddliaceae
Parachlamydiaceae
Sororchlamydiaceae
Chlamydiaceae
outgroup
Tree scale: 0.1

Supplementary fig. 3. *Clavichlamydia salmonicola* is most closely related to other genera in the Chlamydiaceae and the Sororchlamydiaceae. Genome phylogeny using publicly available chlamydial genome sequences as well as Planctomycetes and Verrucomicrobia sequences as an outgroup (Supplementary table 2). Maximum likelihood tree inference was performed with 43 concatenated CheckM single copy marker protein sequences using IQ-TREE (-TESTNEW -PMSF -b 100). Non-parametric bootstrap values are indicated in the tree.

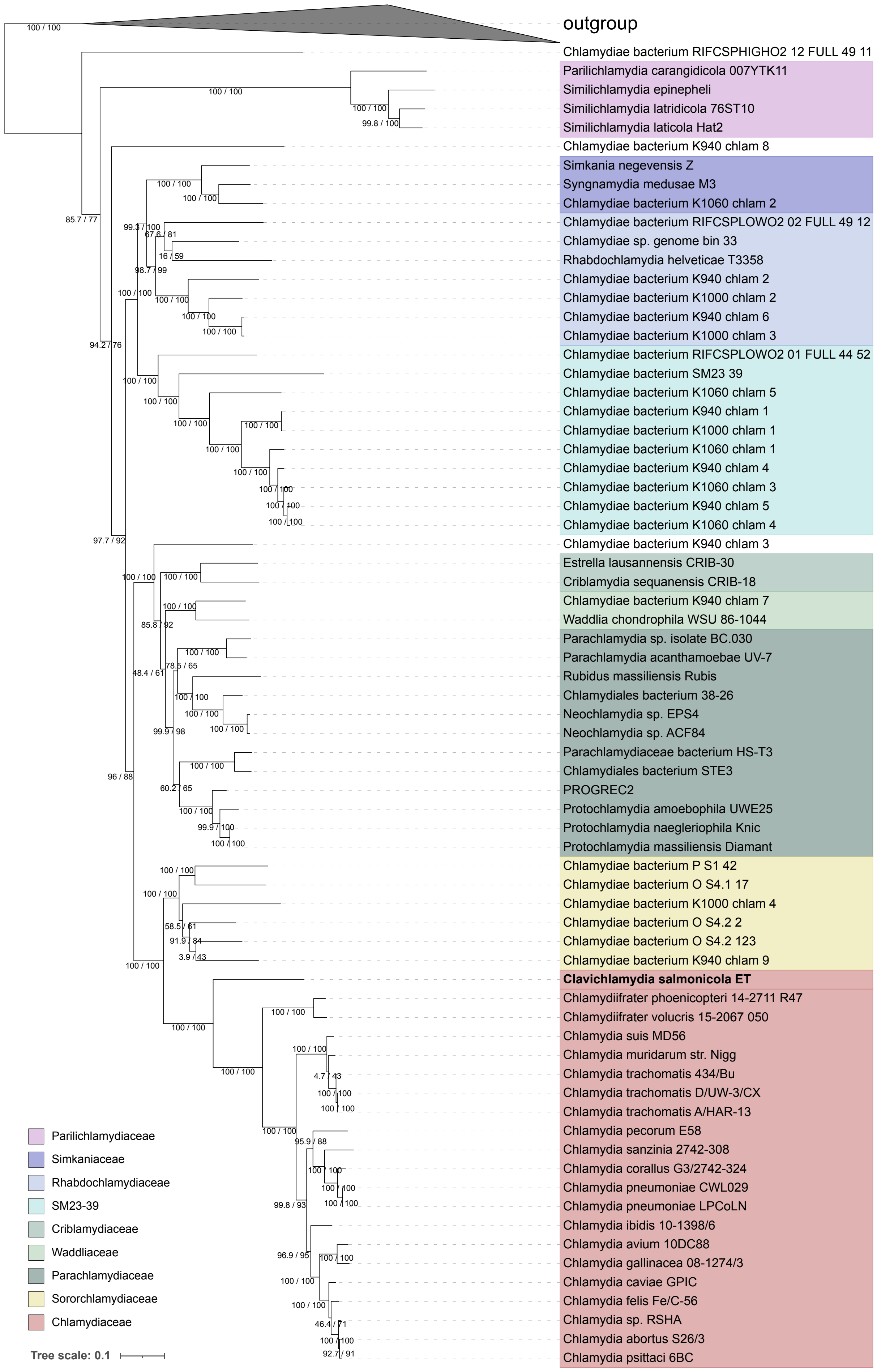
outgroup
Chlamydiae bacterium RIFCSPHIGHO2 12 FULL 49 11
Parilichlamydia carangidicola 007YTK11
Similichlamydia epinepheli
Similichlamydia latridicola 76ST10
Similichlamydia laticola Hat2
Chlamydiae bacterium K940 chlam 8
Simkania negevensis Z
Syngnamydia medusae M3
Chlamydiae bacterium K1060 chlam 2
Chlamydiae bacterium RIFCSPLOWO2 02 FULL 49 12
Chlamydiae sp. genome bin 33
Rhabdochlamydia helveticae T3358
Chlamydiae bacterium K940 chlam 2
Chlamydiae bacterium K1000 chlam 2
Chlamydiae bacterium K940 chlam 6
Chlamydiae bacterium K1000 chlam 3
Chlamydiae bacterium RIFCSPLOWO2 01 FULL 44 52
Chlamydiae bacterium SM23 39
Chlamydiae bacterium K1060 chlam 5
Chlamydiae bacterium K940 chlam 1
Chlamydiae bacterium K1000 chlam 1
Chlamydiae bacterium K1060 chlam 1
Chlamydiae bacterium K940 chlam 4
Chlamydiae bacterium K1060 chlam 3
Chlamydiae bacterium K940 chlam 5
Chlamydiae bacterium K1060 chlam 4
Chlamydiae bacterium K940 chlam 3
Estrella lausannensis CRIB-30
Criblamydia sequanensis CRIB-18
Chlamydiae bacterium K940 chlam 7
Waddlia chondrophila WSU 86-1044
Parachlamydia sp. isolate BC.030
Parachlamydia acanthamoebae UV-7
Rubidus massiliensis Rubis
Chlamydiales bacterium 38-26
Neochlamydia sp. EPS4
Neochlamydia sp. ACF84
Parachlamydiaceae bacterium HS-T3
Chlamydiales bacterium STE3
PROGREC2
Protochlamydia amoebophila UWE25
Protochlamydia naegleriophila Knic
Protochlamydia massiliensis Diamant
Chlamydiae bacterium P S1 42
Chlamydiae bacterium O S4.1 17
Chlamydiae bacterium K1000 chlam 4
Chlamydiae bacterium O S4.2 2
Chlamydiae bacterium O S4.2 123
Chlamydiae bacterium K940 chlam 9
Clavichlamydia salmonicola ET
Chlamydiifrater phoenicopteri 14-2711 R47
Chlamydiifrater volucris 15-2067 050
Chlamydia suis MD56
Chlamydia muridarum str. Nigg
Chlamydia trachomatis 434/Bu
Chlamydia trachomatis D/UW-3/CX
Chlamydia trachomatis A/HAR-13
Chlamydia pecorum E58
Chlamydia sanzinia 2742-308
Chlamydia corallus G3/2742-324
Chlamydia pneumoniae CWL029
Chlamydia pneumoniae LPCoLN
Chlamydia ibidis 10-1398/6
Chlamydia avium 10DC88
Chlamydia gallinacea 08-1274/3
Chlamydia caviae GPIC
Chlamydia felis Fe/C-56
Chlamydia sp. RSHA
Chlamydia abortus S26/3
Chlamydia psittaci 6BC
Parilichlamydiaceae
Simkaniaceae
Rhabdochlamydiaceae
SM23-39
Criblamydiaceae
Waddliaceae
Parachlamydiaceae
Sororchlamydiaceae
Chlamydiaceae
Tree scale: 0.1

Supplementary fig. 4. Initial maximum likelihood tree inference serving as guide tree for the posterior mean site frequency (PMSF) modeled tree including ultrafast bootstrap values and SH-like approximate likelihood ratio test values (IQ-TREE -TESTNEW -bnni 1000 -alrt 1000), respectively. The phylogenetic tree is rooted with sequences from Planctomycetes and Verrucomicrobia as an outgroup.

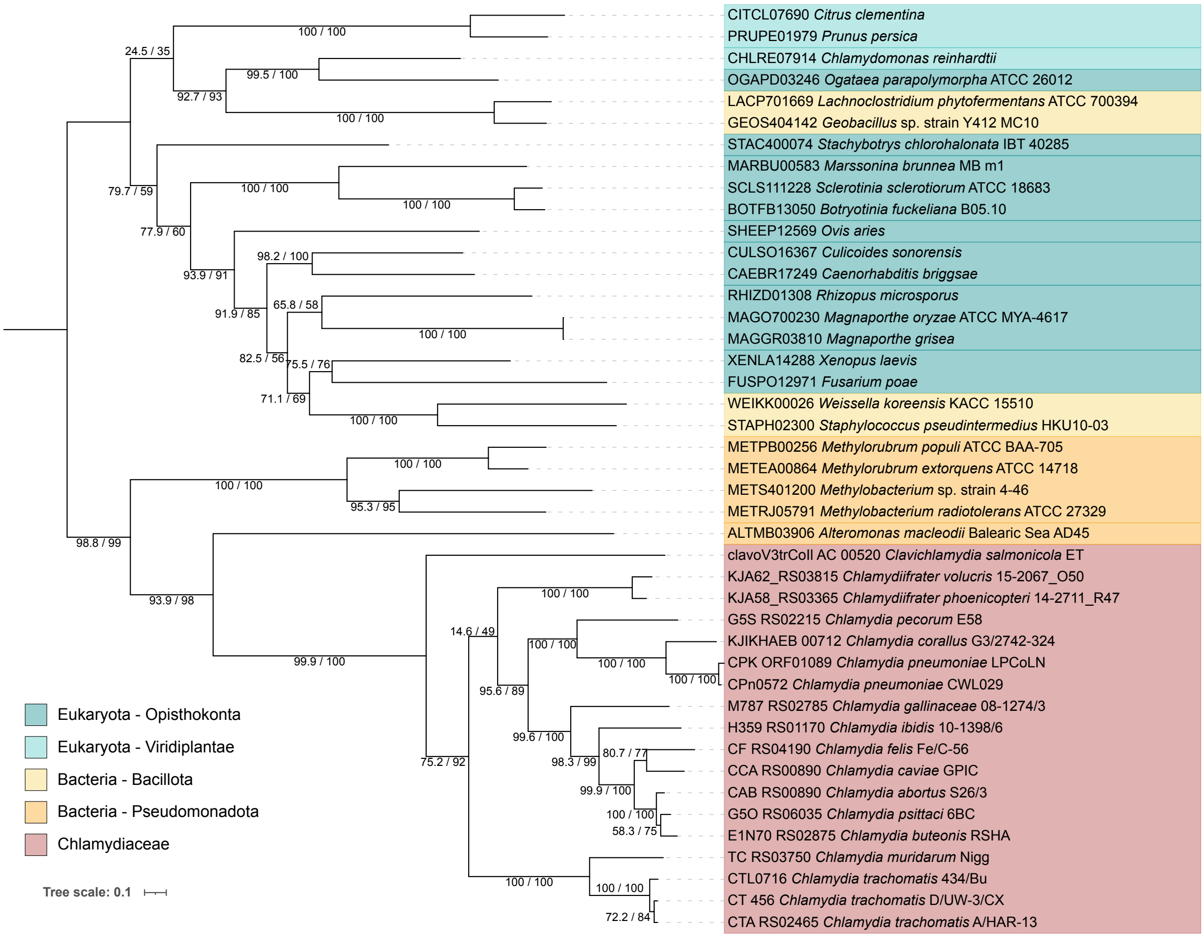

Supplementary fig. 5. TarP phylogeny including protein sequences from Chlamydiaceae and OMA orthologs (Altenhoff, 2021) indicates horizontal acquisition of this gene from Pseudomonadota in the last common Chlamydiaceae ancestor and subsequent maintenance and diversification within the family. Maximum likelihood tree inference using the PMB+F+G4 substitution model (IQ-TREE -TESTNEW -bnni -bb 1000 -alrt 1000). The phylogenetic tree is midpoint rooted. Species are color coded according to their taxonomy. Ultrafast bootstrap support values and SH-like approximate likelihood ratio test values are indicated, respectively.

3. Supplementary references

Altenhoff A, Train C-M, Gilbert KJ, Mediratta I, Mendes de Farias T, Moi D, Nevers Y, Radoykova H-S, Rossier V, Vesztrocy AW, Glover NM, Dessimoz C (2021) OMA orthology in 2021: website overhaul, conserved isoforms, ancestral gene order and more. Nucleic Acids Res 49: D373-D379.

Haider S, Collingro A, Walochnik J, Wagner M, Horn M (2008) *Chlamydia*-like bacteria in respiratory samples of community-acquired pneumonia patients. FEMS Microbiology Letters 281: 198-202.

Juretschko S, Timmermann G, Schmid M, Schleifer KH, Pommerening-Roser A, et al. (1998) Combined molecular and conventional analyses of nitrifying bacterium diversity in activated sludge: *Nitrosococcus mobilis* and *Nitrospira*-like bacteria as dominant populations. Appl Environ Microbiol 64: 3042-3051.

Loy A, Schulz C, Luecker S, Schopfer-Wendels A, Stoecker K, et al. (2005) 16S rRNA gene-based oligonucleotide microarray for environmental monitoring of the betaproteobacterial order "*Rhodocyclales*". Appl Environ Microbiol 71: 1373-1386.

Schmitz-Esser S, Toenshoff ER, Haider S, Heinz E, Hoenninger VM, Wagner M, Horn M (2008) Diversity of bacterial endosymbionts of environmental *Acanthamoeba* isolates. Appl Environ Microbiol 74: 5822-5831.
